# [Corrigendum] *Ampelopsis japonica* ethanol extract suppresses migration and invasion in human MDA‑MB‑231 breast cancer cells

**DOI:** 10.3892/mmr.2025.13527

**Published:** 2025-04-10

**Authors:** Kyoung Jin Nho, Jin Mi Chun, Dong-Seon Kim, Ho Kyoung Kim

Mol Med Rep 11: 3722–3728, 2015; DOI: 10.3892/mmr.2015.3179

Subsequently to the publication of the above paper, an interested reader drew to the authors’ attention that, in Fig. 1C on p. 3724, the ‘12 h, 25 μg/ml EAJ’ and ‘12 h, 50 μg/ml EAJ’ panels (second and third panels from the left on the top row)appeared to contain matching data, such that they were derived from the same original source where these panels were intended to show the results from differently performed experiments. The authors have re-examined their original data, and realize that the ‘12 h, 50 μg/ml EAJ’ panel was inadvertently duplicated in the figure.

The revised version of Fig. 1, now containing the correct data for the ‘12 h, 25 μg/ml EAJ’ panel in Fig. 1C, is shown below. Note that this error did not grossly affect either the results or the overall conclusions reported in this study. All the authors agree with the publication of this corrigendum, and are grateful to the Editor of *Molecular Medicine Reports* for allowing them the opportunity to publish this. They also wish to apologize to the readership of the Journal for any inconvenience caused.

## Figures and Tables

**Figure 1.**
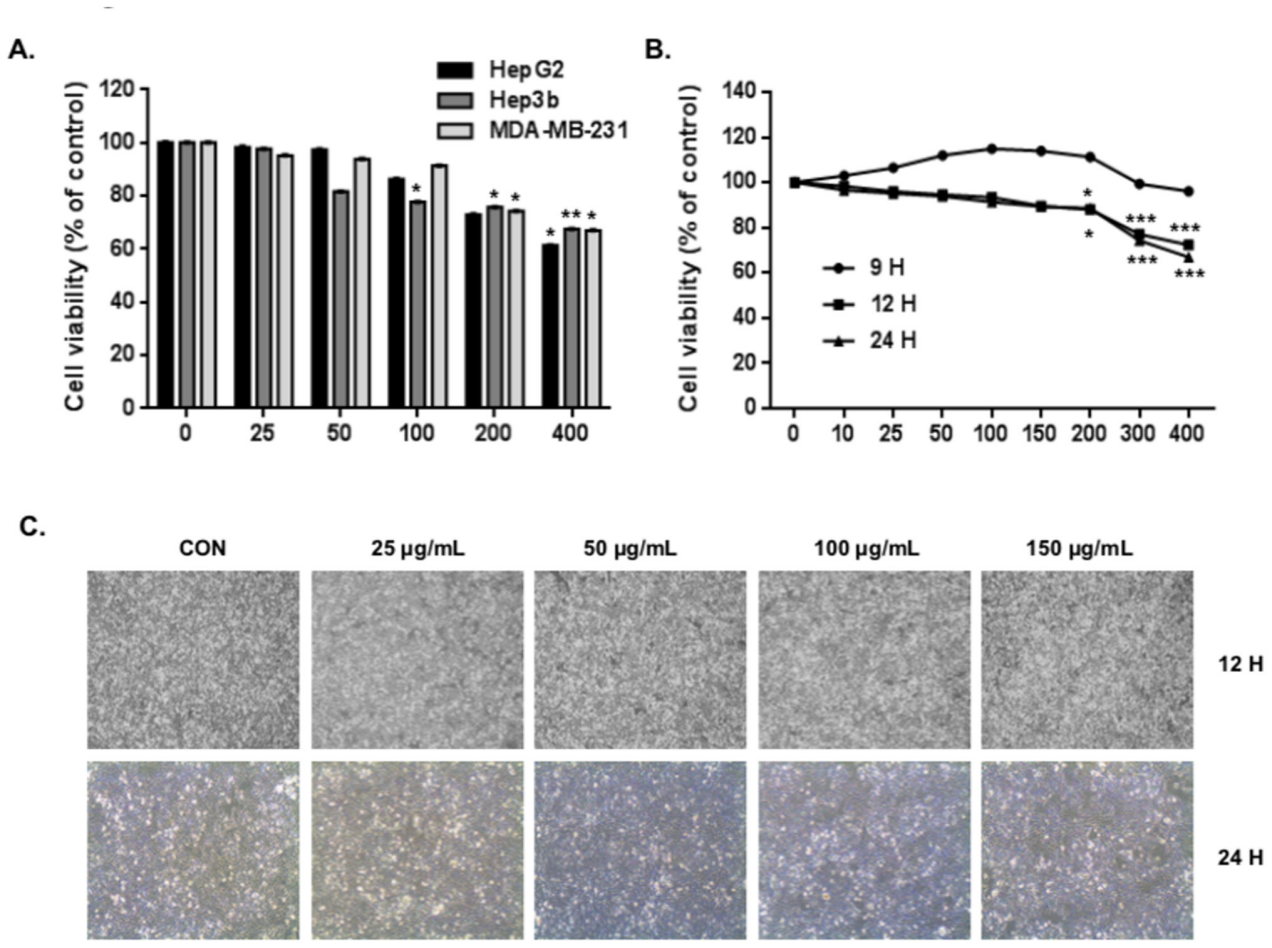
EAJ reduces the viability of MDA-MB-231 breast cancer cells. (A) HepG2, Hep3b and MDA-MB-21 cells were exposed to various concentrations of EAJ (0, 25, 50, 100, 200 or 400 μg/ml) for 24 h. (B) MDA-MB-231 cells were exposed to the indicated concentrations of EAJ for 9, 12 or 24 h. Cell viability was then assessed using a Cell Counting kit-8 assay. Values are presented as the mean ± standard deviation (n=3). *P<0.05, **P<0.01 and ***P<0.001, vs. control. (C) Cell morphology was examined under an inversion microscope following treatment of MDA-MB-231 cells with the indicated concentrations of EAJ magnification, ×200). EAJ, *Ampelopsis japonica* ethanol extract; CON, control (0 μg/ml EAJ).

